# Acute myocardial infarction in an untreated patient with acute myeloid leukemia

**DOI:** 10.1002/ccr3.8601

**Published:** 2024-03-13

**Authors:** Doreen Kamoga, Sai Desikan, Raman Desikan, Jackson Musuuza

**Affiliations:** ^1^ Department of Internal Medicine White River Health Batesville Arkansas USA; ^2^ MD Anderson Cancer Center Houston Texas USA; ^3^ Department of Hematology/Oncology White River Health Batesville Arkansas USA

**Keywords:** acute leukemia, hypercoagulability, myocardial infarction, thrombosis

## Abstract

**Key Clinical Message:**

Acute leukemia, particularly AML, is closely associated with thrombotic events, driven by complex factors like coagulation system changes, endothelial dysfunction, and leukemic cell interactions with the vascular system. Certain chemotherapy drugs can exacerbate the prothrombotic state. Understanding these dynamics is crucial for effective thromboprophylaxis in carefully selected patients with leukemia.

**Abstract:**

Thrombosis is a significant complication of acute leukemia. Thrombotic events mostly occur at diagnosis or during induction therapy. Here we report the occurrence of myocardial infarction (MI) before initiation of therapy, in a patient with acute myeloid leukemia not otherwise specified (AML NOS) who had no other significant risk factors for coronary artery disease. The occurrence of MI in this patient limited the choice of induction therapy and resulted in mortality. We discuss the pathogenesis and risk factors associated with increased thrombosis in AML and advocate for risk‐adapted thromboprophylaxis in this patient population.

## 1 INTRODUCTION

Venous thromboembolism (VTE) has been appreciated in cancers including leukemia. Arterial thromboembolism (ATE), such as myocardial infarction (MI) and stroke, is also prevalent. Increased risk of venous and arterial thrombosis at presentation has been directly associated with underlying leukemia.^1^ The American Society of Clinical Oncology (ASCO) Clinical Practice Guideline Update 2019 recommends that hospitalized patients with active malignancy without additional risk factors may be offered pharmacologic thromboprophylaxis in the absence of bleeding or other contraindications.^2^ Utility of and risks associated with anticoagulants have been studied extensively in VTE, but not in ATE. There is paucity of data addressing the management of ATE in patients with cancer. We report the occurrence of MI before initiation of therapy, in a patient with AML with no other significant risk factors for coronary artery disease (CAD). We discuss the pathogenesis and risk factors for increased thrombosis, and advocate for risk‐adapted thromboprophylaxis in patients with AML.

The purpose of this case report is not to imply causation between AML and MI, but to raise awareness about other possible serious hypercoagulability‐related events in AML such as MI. Our goal is to encourage clinicians to institute early preventive measures when managing patients with AML.

## 2 CASE HISTORY/EXAMINATION/REPRESENTATION

**FIGURE 1:**
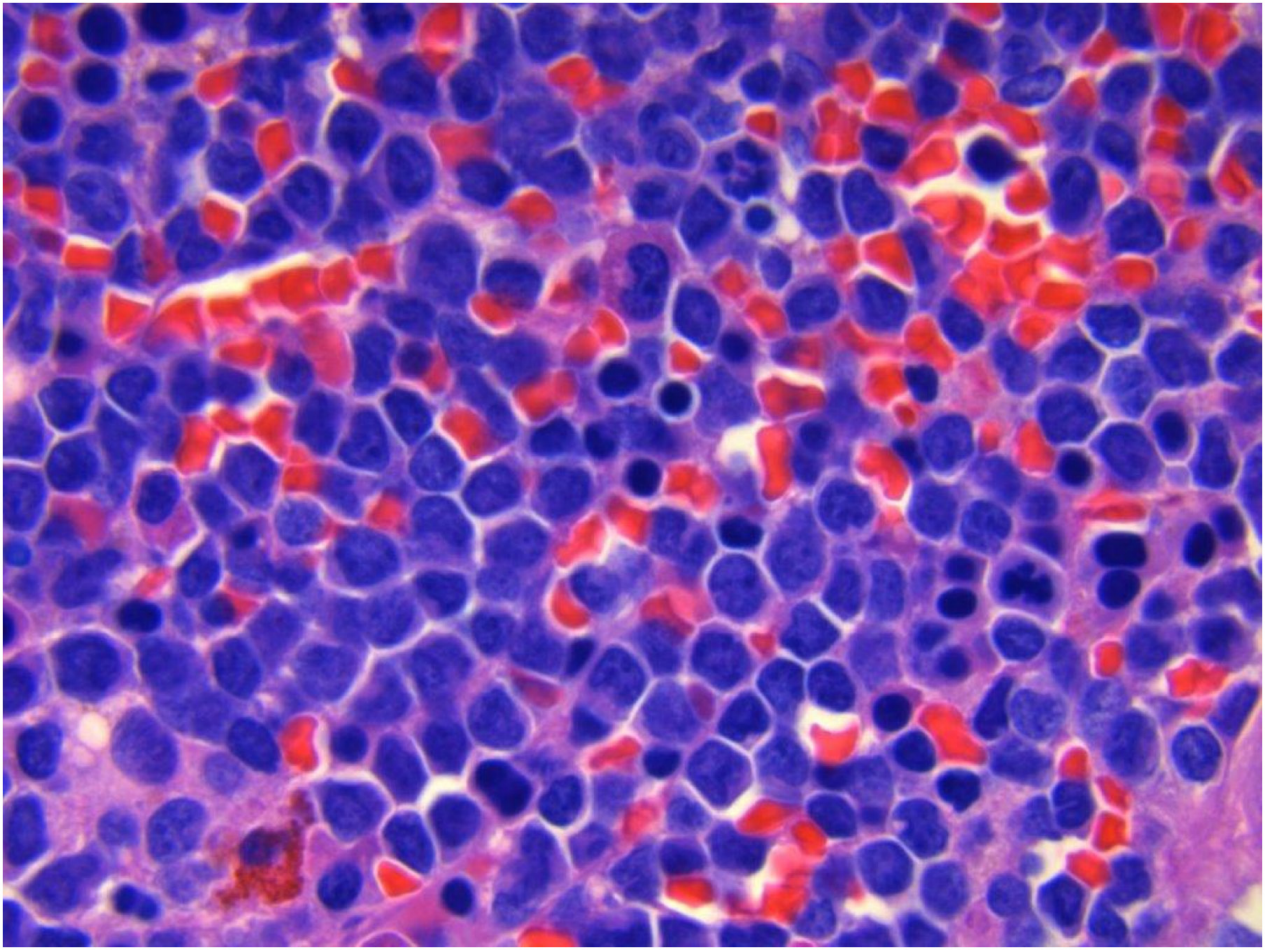
A bone marrow biopsy showing hypercellular marrow with 90% blasts.

**FIGURE 2:**
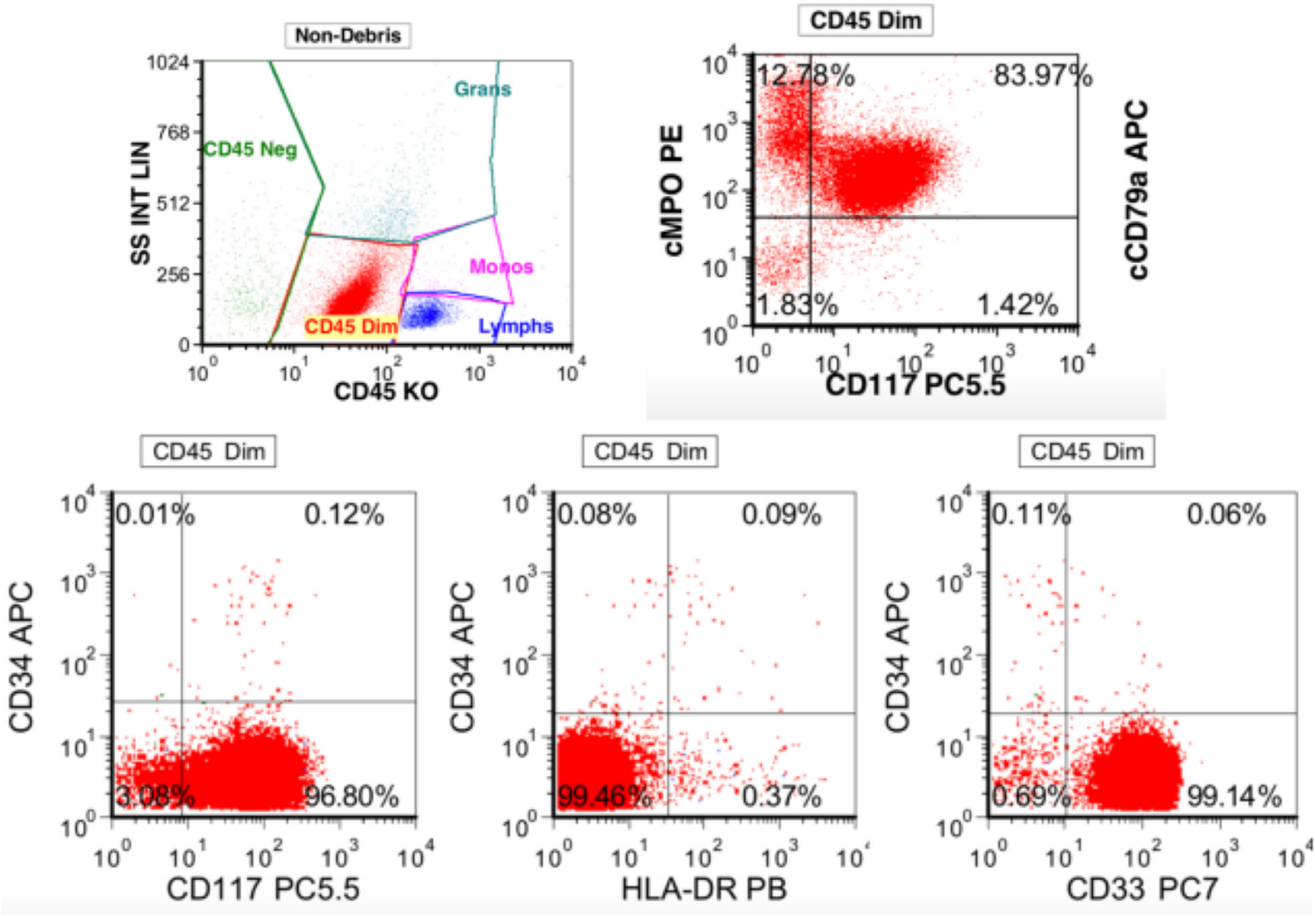
Flow cytometric phenotype positive for CD33, CD117 and negative for CD34 and HLA DR consistent with APL.

A 56‐year‐old white male without significant medical history was evaluated at a rural hospital in USA for neutropenia (white cell count of 1.7 × 10^9^/L and absolute neutrophil count of 0.795 × 10^9^/L). The bone marrow was hypercellular for age (90% cellularity) parked with sheets of immature cells or blasts (Figure 1). Flow cytometric phenotype was positive for CD33, CD117 and negative for CD34 and HLA DR (Figure 2). Fluorescent insitu hybridization (FISH) was negative for translocation (15;17). Karyotype analysis was normal, and FISH revealed no abnormality and negative for variant translocations. Mutation analysis revealed FLT3 internal tandem duplication (ITD) and NPM1 mutations, CEBPA mutation was not detected. Overall, the morphologic, and cytogenetic analysis was diagnostic of acute myeloid leukemia not otherwise specified (AML NOS). The patient was asked to proceed to a referral University Hospital for administration of induction chemotherapy. Unfortunately, he did not initially comply with this recommendation. Two days later the patient was hospitalized with worsening chest discomfort, nausea, and emesis. ECG findings and elevated cardiac enzymes [CK 23.6 μkat/L (0.65–5.13), CK‐MB 137 μg/L (0–6), and troponin 15.14 μg/L (0–0.034)] were consistent with ST segment elevation MI. The patient underwent thrombectomy, angioplasty, and stenting of left anterior descending artery (LAD) and diagonal arteries. At this time, the patient was transferred to a University Hospital for induction therapy for leukemia.

## 3 METHODS

### 3.1 Differential diagnosis

Differential diagnoses for chest pain in this patient included acute pericarditis, Prinzmetal's angina, and stress induced/Takotsubo cardiomyopathy. All these can lead to ST elevations on the ECG. The main distinguishing feature of MI is the presence of coronary arteries occlusion on cardiac catheterization.

### 3.2 Investigations

Hemoglobin (7.82 mmol/L) and platelets (168 × 10^9^/L) were preserved. Peripheral smear did not reveal immature cells. Antinuclear antibody, rheumatoid factor, serology for hepatitis, and HIV were negative. Flow cytometry to evaluate large granular lymphocytosis revealed abnormal clone: 3% of cells were positive for CD117 and CD33. Lipid profile showed a slight elevation of HDL cholesterol [0.83 mmol/L (1.01–1.58)]. Total cholesterol [3.72 mmol/L (2.77–5.18)], LDL cholesterol [2.41 mmol/L (0–2.56)], and triglycerides (1.11 mmol/L) were normal. Hemoglobin A1C was normal at 5.5%, PT was slightly elevated (13.9 s) with INR of 1.3, PTT was normal, and D‐dimer was significantly elevated at 20 mgFEU/L (0–0.48). Protein C, S, and antithrombin III levels were normal. Homocysteine was elevated at 21.7 μmol/L (< 11.4 μmol/L). Factor V Leiden mutation was not detected. Peripheral smear revealed more than 90% blasts.

### 3.3 Treatment

The patient received induction therapy for leukemia. Due to significant cardiac impairment, he received Azacitidine for 10 days. The MI was managed with AngioJet thrombectomy and placement of a 3.5 × 38‐mm stent in the proximal and upper‐to‐mid left anterior descending artery.

## 4 CONCLUSION AND RESULTS

From the University Hospital, the patient was discharged with close follow‐up and transfusion support by his hematologist. Platelet and packed red cell transfusions were administered as indicated. On the last evaluation by his oncologist, 2 weeks after discharge from the University Hospital, an increase in circulating blasts was noted. He was to be re‐evaluated in the University Hospital for further chemotherapy. Unfortunately, he had sudden death at home the following day after the above oncologist follow‐up.

## 5 DISCUSSION

We present a case of acute ST segment elevation MI in a patient with AML. Other than male sex and history of fatal MI in his father at age 56, the patient had no significant risk factors for CAD such as hypertension, hypercholesterolemia, diabetes mellitus, current tobacco use, and obesity.^3^ The patient's family history of MI (usually mediated through familial hypercholesterolemia), without other major CAD risk factors, is less likely to have contributed to the occurrence of MI in this patient. Clinical diagnosis of familial hypercholesterolemia requires an LDL cholesterol elevation up to 4.91 mmol/L or greater and either a first‐degree relative with an LDL cholesterol level of 4.91 mmol/L or greater or with known premature CAD occurring before age 55 years in males.^4^ In our patient, thrombophilia evaluation was normal except for elevated homocysteine levels, which can be observed in multiple malignancies including leukemia.^5^ The absence of thrombophilia and the presence of a temporal relationship between increased blast count and the occurrence of MI strengthens the association between the MI the increased thrombosis due to acute leukemia.

Acute leukemia is associated with increased risk of venous and arterial thrombosis. In one study, the incidence of venous and arterial thrombosis among patients with acute leukemia was 80% and 20%, respectively.^6^ All arterial thrombosis was observed among patients with AML.^6^ A higher incidence of venous and arterial thrombosis in promyelocytic leukemia has also been reported in other studies.^7^ The hypercoagulable state in leukemia occurs through several direct and indirect mechanisms.^8^ Hypercoagulability in leukemia stems from factors such as expression of tissue factor or tissue factor–bearing microparticles that can lead to systemic thrombi. Procoagulant elevation is more prominent in acute promyelocytic leukemia (APL) compared to other forms of leukemias.^9^ Aberrant surface expression of Podoplanin, a platelet aggregation agonist could also contribute to the procoagulable state.^10^

Promyeloblasts in patients with APL disproportionately express certain cytokines such as TNF‐α, IL‐1β, and IL‐6. IL‐1β and TNF‐α augment the activity of tissue factor and plasminogen activator inhibitor‐1 (PAI‐1), all of which may increase the propensity for hypercoagulation in leukemia.^11^ In addition, TNF‐ α can downregulate production of thrombomodulin (up to less than 80%) increasing hypercoagulability propensity.^10^

As we discuss above, the pathogenesis of hypercoagulability in patients with acute leukemia and acute MI is not clearly defined, but it is related to disseminated intravascular coagulation (DIC).

Thrombosis in acute leukemia is associated with increased DIC markers such as elevated D‐dimer levels, but this is more prominently seen in APL.^12^ The pathogenesis of thrombosis is distinctly different from coronary thrombosis in AMI. With advancement in differentiated therapy, DIC has been less prevalent; however, there remains an increased risk of thrombosis.

Treatment of thrombosis in AML can mirror the observations in DIC and involves administration of antithrombin III and recombinant human‐soluble thrombomodulin (rhTM). These work on the premise of targeting the hemostatic system. Antithrombin III, a serine protease inhibitor acts by inhibiting thrombin and activated factor X. In a clinical trial by Saito et al., thrombomodulin was shown to resolve DIC and improve the overall clinical course of bleeding symptoms.^13^ Such therapies could be used for treatment of thrombosis in patients with acute leukemia and acute MI.

## AUTHOR CONTRIBUTIONS

**Doreen Kamoga:** Conceptualization; methodology; project administration. **Sai Desikan:** Conceptualization; methodology; writing – review and editing. **Raman Desikan:** Conceptualization; methodology; resources. **Jackson Musuuza:** Conceptualization; methodology; writing – original draft; writing – review and editing.

## FUNDING INFORMATION

None of the authors received any specific grant from any funding. agency in the public, commercial, or not‐for‐profit sectors.

## CONSENT

Written informed consent was obtained from the patient's next of kin to publish this report in accordance with the journal's patient consent policy.

## Data Availability

Data available on request from the authors.
